# Proteome-*pI* 2.0: proteome isoelectric point database update

**DOI:** 10.1093/nar/gkab944

**Published:** 2021-10-28

**Authors:** Lukasz Pawel Kozlowski

**Affiliations:** Institute of Informatics, Faculty of Mathematics, Informatics, and Mechanics, University of Warsaw, Warsaw, Mazovian Voivodeship 02-097, Poland

## Abstract

Proteome-*pI* 2.0 is an update of an online database containing predicted isoelectric points and p*K*_a_ dissociation constants of proteins and peptides. The isoelectric point—the pH at which a particular molecule carries no net electrical charge—is an important parameter for many analytical biochemistry and proteomics techniques. Additionally, it can be obtained directly from the p*K*_a_ values of individual charged residues of the protein. The Proteome-*pI* 2.0 database includes data for over 61 million protein sequences from 20 115 proteomes (three to four times more than the previous release). The isoelectric point for proteins is predicted by 21 methods, whereas pKa values are inferred by one method. To facilitate bottom-up proteomics analysis, individual proteomes were digested *in silico* with the five most commonly used proteases (trypsin, chymotrypsin, trypsin + LysC, LysN, ArgC), and the peptides’ isoelectric point and molecular weights were calculated. The database enables the retrieval of virtual 2D-PAGE plots and customized fractions of a proteome based on the isoelectric point and molecular weight. In addition, isoelectric points for proteins in NCBI non-redundant (nr), UniProt, SwissProt, and Protein Data Bank are available in both CSV and FASTA formats. The database can be accessed at http://isoelectricpointdb2.org.

## Graphical Abstract

**Graphical Abstract:**
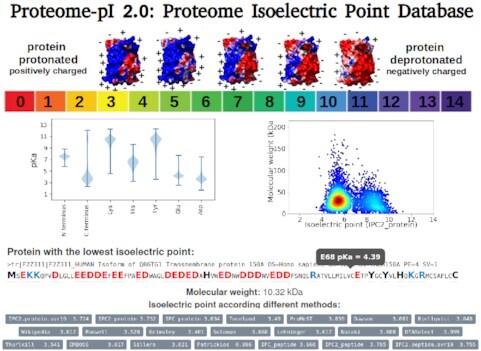
Proteome-pI 2.0 database: 61,329,034 proteins from 20,115 proteomes with isoelectric point predicted using 21 methods; 5.38B dissociation constant (pKa) predictions; 9.58B in silico digested peptides (Trypsin, Chymotrypsin, Trypsin+LysC, LysN, ArgC).

## INTRODUCTION

**Figure 1.**
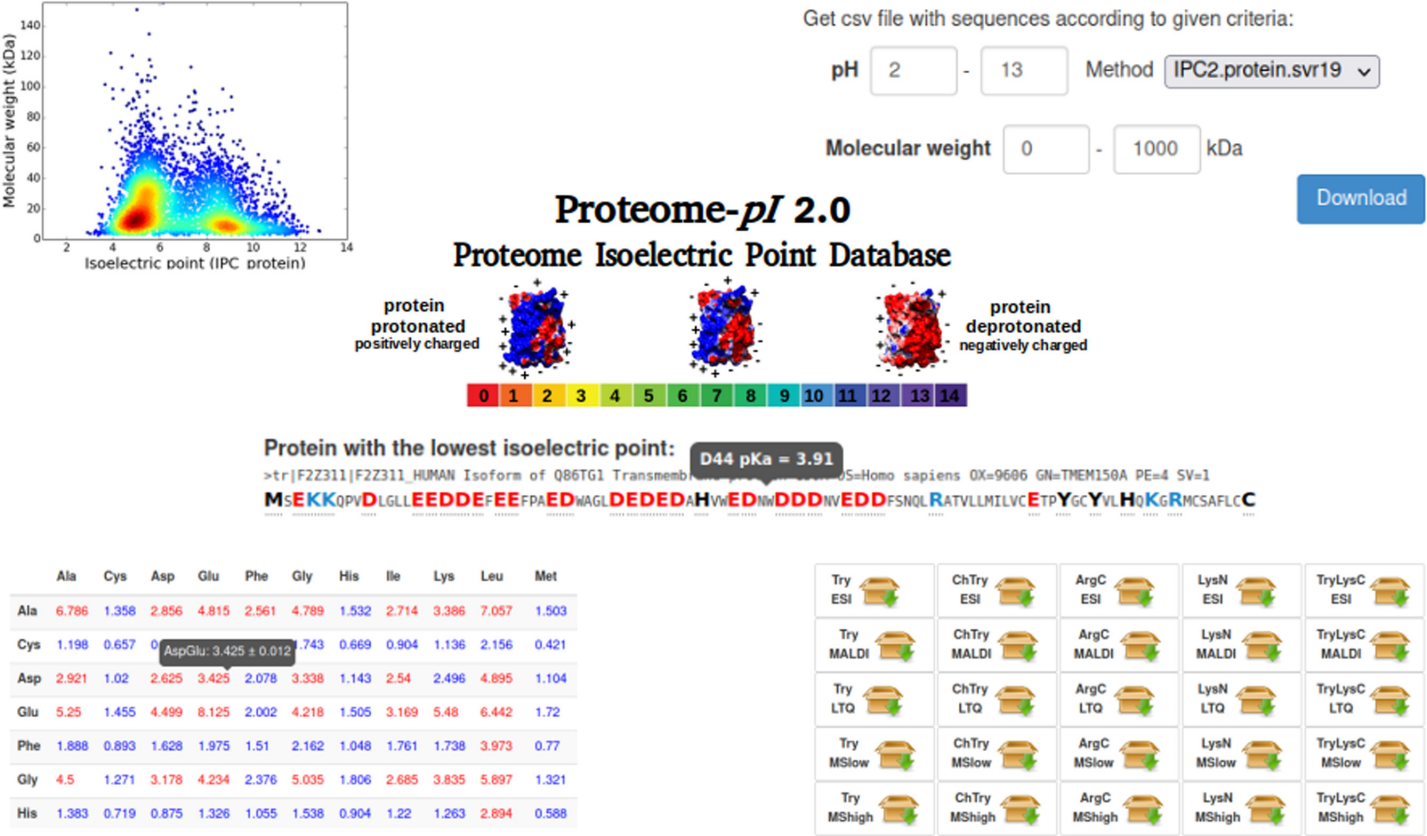
An overview of the Proteome-*pI* 2.0 database. Isoelectric points and molecular weights for individual proteins from 20 115 proteomes are visualized on virtual 2D PAGE plots (top left) and can be retrieved according to the predictions from one of 21 algorithms (top right). The data for individual proteins are accompanied by dissociation constant (p*K*_a_) predictions (middle). The proteomes are digested *in silico* by one of the five most commonly used proteases (trypsin, chymotrypsin, trypsin + LysC, LysN, ArgC) (bottom right). Additionally, auxiliary statistics are provided (e.g. di-amino acid frequencies) (bottom left).

The charge of a protein is one of its key physicochemical characteristics and is related to the p*K*_a_ dissociation constant (p*K*_a_ is a quantitative measure of the strength of an acid in solution). For proteins and peptides, the ionizable groups of seven charged amino acids should be considered: glutamate (γ-carboxyl group), cysteine (thiol group), aspartate (β-carboxyl group), tyrosine (phenol group), lysine (ε-ammonium group), histidine (imidazole side chains), and arginine (guanidinium group) (1). Taken together, the p*K*_a_ values of all charged groups can be used to calculate the overall charge of the molecule in any pH or to estimate the isoelectric point (*pI*, IEP), that is, the pH at which there is an equilibrium of positive and negative charges and therefore the total net charge of the molecule is equal to zero (2). Both p*K*_a_ and isoelectric point estimates have been used in numerous techniques, such as two-dimensional gel electrophoresis (2D-PAGE) (3,4), crystallization (5), capillary isoelectric focussing (6), and mass spectrometry (MS) (7,8). It should be stressed that experimental measurements of p*K*_a_ values [PKAD database (9)] and isoelectric point [SWISS-2DPAGE (10)] are very limited (a few thousand records at most), but there are many computational methods that can be used to predict these features. In this work, I present a major update of the original Proteome-*pI* database (Figure 1) (11). The following changes have been introduced:

- the number of proteomes included has been increased four-fold (from 5029 to 20 115);
- new algorithms for isoelectric point prediction have been added (21 algorithms in total);
- the prediction of p*K*_a_ dissociation constants for over 61 million proteins have been included;
- the prediction of isoelectric point for *in silico* digests of proteomes with the five most commonly used proteases (trypsin, chymotrypsin, trypsin + LysC, LysN, ArgC) have been added.

## MATERIALS AND METHODS

### Datasets

Proteome-*pI* 2.0 is based on UniProt (12) reference proteomes (2021_03 release) and contains over 61 million protein sequences coming from 20 115 model organisms (Table 1 and Supplementary Table S1). The data are divided according to the major kingdoms of the tree of life and include splicing variants for eukaryotic organisms. Additionally, the isoelectric point is predicted for the most commonly used protein sequence databases, such as the entire UniProt TrEMBL with 219 million sequences (12), SwissProt with 561 000 proteins (13,14), NCBI nr (non-redundant) with 409 million sequences (15), and Protein Data Bank with 601 000 protein chains (16).

**Table 1.** General statistics of the Proteome-*pI* 2.0 database (20 115 proteomes with 61 329 034 proteins in total)

	Number of proteomes	Total number of proteins	Mean number of proteins (±SD)	Mean size of proteins (±SD)	Mean mw of proteins (±SD)
Viruses	10 064	518 140	51 ± 85	237 ± 300	26.6 ± 33.2
Archaea	331	767 951	2320 ± 1263	278 ± 211	30.6 ± 23.1
Bacteria	8108	30 290 647	3736 ± 1785	320 ± 246	35.1 ± 26.8
Eukaryote	1612	29 752 296	18457 ± 16804	467 ± 471	52.1 ± 52.4
Eukaryote (major)	1612	25 437 198	15780 ± 11138	438 ± 420	48.8 ± 46.7
Eukaryote (minor)	637	4 315 098	6774 ± 14244	638 ± 676	71.2 ± 75.4

mw, molecular weight in kDa; mean size in amino acids. For more statistics, see Supplementary Table S1. ‘Major’ and ‘minor’ refer to splicing isoforms of proteins used for calculation of the statistics.

### Predictions for proteins

Each proteome is analysed by various methods. The prediction of the isoelectric point is currently performed using 21 methods (including four new ones), which can be grouped into two categories. The simplest methods of isoelectric point prediction are based on experimentally derived p*K*_a_ sets and the Henderson–Hasselbach equation: Patrickios (17), Solomons (18), Lehninger (19), EMBOSS (20), Dawson (21), Wikipedia (p*K*_a_ values as presented in Wikipedia page in 2005), Toseland (22), Sillero (23), Thurlkill (24), Rodwell (25), DTASelect (26), Nozaki (27), Grimsley (28), Bjellqvist (29) [whose method was implemented as ExPASy ‘Compute pI/Mw Tool’ (30)] and ProMoST (31). The second group includes methods that are based on machine learning [IPC_protein, IPC_peptide, IPC2_protein, IPC2_peptide, IPC2.peptide.svr19, and IPC2.protein.svr (32,33)]. Moreover, in Proteome-*pI* 2.0, a completely new category of predictions has been introduced, namely the prediction of p*K*_a_ dissociation constants. In this case, only one algorithm is used [IPC2.pKa (33)], as other methods for p*K*_a_ prediction are prohibitively slow and additionally require structural data (not available in Proteome-*pI*) (34–37).

### Predictions for peptides

To facilitate bottom-up mass spectrometry analysis, *in silico* proteolytic digestion of proteins by the five most commonly used proteases (trypsin, chymotrypsin, trypsin + LysC, LysN, ArgC) has been introduced (38). The proteolytic products (i.e. peptides) are treated as the surrogates of the parent proteins for further qualitative or quantitative analysis. The proteases generally cleave proteins at specific amino acid residue sites, but digestion is frequently incomplete (missed cleavage sites are widespread). To predict proteolysis, the Rapid Peptides Generator (RPG) program was used (with a 1.4% miscleavage rate) (39). The resulting five datasets are further categorized according to the molecular mass of the peptides (Figure 1 and Supplementary Table S2): ESI Ion Trap (600–3500 Da), LTQ Orbitrap (600–4000 Da), MALDI TOF/TOF (750–5500 Da), MS low (narrow range of mass, 800–3500 Da), and MS high (wide range of mass, 600–5500 Da) (35). Finally, for the resulting peptides, the isoelectric point is predicted.

## RESULTS

A single results page for Proteome-*pI* displays a comprehensive overview of the complete proteome (one from 20 115 model organisms). The isoelectric point predictions for all proteins (including splicing isoforms or alternative sequences) are available, together with a virtual 2D-PAGE plot. The user can retrieve customized datasets according to specified isoelectric point and molecular mass ranges. Extreme examples (proteins with minimal and maximal isoelectric point predictions) are then presented. The information is complemented with plots depicting global isoelectric point and p*K*_a_ predictions according different methods (Supplementary Figure S1). In the next panel, the user can find *in silico* digests of the whole proteome with trypsin, chymotrypsin, trypsin + LysC, LysN and ArgC proteases suitable for different mass spectrometry machines, such as the ESI Ion Trap (600–3500 Da), LTQ Orbitrap (600–4000 Da), MALDI TOF/TOF (750–5500 Da), MS low (narrow range of mass, 800–3500 Da), and MS high (wide range of mass, 600–5500 Da). This can result in a huge number of potential peptides (e.g. for human proteins, trypsin digests can exceed two million peptides; Supplementary Table S2). At the bottom, general statistics such as amino acid and di-amino acid frequencies can be found. Additionally, each page is interconnected to external databases, such as UniProt and NCBI Taxonomy.

Furthermore, Proteome-*pI* 2.0 provides global analyses related to the distribution of molecular weight and isoelectric points across kingdoms, or amino and di-amino acid statistics (Table 2 and Supplementary Table S3). Such data can be useful for high-throughput analysis of specific taxons, such as plants (40), fungi (41) or groups of interacting proteins (42).

**Table 2.** Amino acid frequency for the kingdoms of life in the Proteome*-pI* 2.0 database

Kingdom	Ala	Cys	Asp	Glu	Phe	Gly	His	Ile	Lys	Leu	Met	Asn	Pro	Gln	Arg	Ser	Thr	Val	Trp	Tyr	Total amino acids
Viruses	7.81	1.29	6.20	6.46	3.91	6.72	1.96	6.05	6.24	8.28	2.51	4.99	4.25	3.62	5.31	6.47	6.14	6.66	1.42	3.71	122 870 810
Archaea	8.95	0.90	w7.00	7.94	3.65	7.84	1.86	6.03	4.18	9.11	2.14	3.36	4.36	2.48	5.83	6.12	5.84	8.16	1.06	3.18	213 285 886
Bacteria	10.64	0.90	5.67	6.06	3.76	8.01	2.08	5.52	4.22	10.12	2.31	3.35	4.82	3.49	6.18	5.75	5.58	7.42	1.31	2.81	9 693 905 784
Eukaryota	7.38	1.85	5.34	6.55	3.79	6.35	2.50	4.94	5.64	9.38	2.27	4.13	5.56	4.27	5.71	8.45	5.56	6.24	1.24	2.81	13 901 635 566
All	8.72	1.46	5.49	6.36	3.78	7.04	2.32	5.19	5.05	9.67	2.29	3.81	5.24	3.94	5.90	7.33	5.57	6.74	1.27	2.81	23 931 698 046

Similar statistics for the 20 115 individual proteomes included in Proteome-*pI* are available online on separate subpages. Additionally, the online version of the table http://isoelectricpointdb2.org/statistics.html is accompanied by an error estimated with 1000 bootstraps. For di-amino acid frequencies, see Supplementary Table S3.

## DISCUSSION

The Proteome-*pI* 2.0 database update is a significant improvement upon the previous version, both quantitatively (covering more proteomes and using more algorithms) and qualitatively (including peptide digests and p*K*_a_ predictions). Nevertheless, apart from the technical extension of the database (analysing more organisms), it is always worth checking how the addition of new data may have affected some global conclusions drawn from the data available at the time of evaluation.

For instance, one of the scientifically important by-products of creating Proteome-*pI* was the observation that the isoelectric points and molecular weights of proteins in different kingdoms vary considerably. For example, Archaea have the smallest proteins (except for viruses), but the isoelectric point of the proteome can differ greatly among individual species. This may be because Archaea are known for living in extreme environments (e.g. low or high pH), which affects the range of isoelectric point in their proteomes. In 2016, when the first version of the database was created, only 135 Archaeal organisms were included, whereas in the current version we have 331 such proteomes. Careful comparison of Figure 2 from Kozlowski (11) with Supplementary Figure S2 shows that indeed the trend is following an analysis of more Archaea, highlighting how unique and diverse these organisms can be in terms of their proteins’ charge (see also Supplementary Figure S1).

Similarly, many statistics calculated previously have been repeated on the larger dataset, using a new version of a proteome or extending the calculation from the statistical perspective. For instance, two auxiliary statistics that Proteome-*pI* provides are amino and di-amino acid frequencies for whole proteomes. In the current version, we added error estimates (with × 100 bootstrapping at the protein level) to assess the possible variability of the calculations. This is not a purely technical aspect, as our knowledge about what constitutes the proteome of a given organism changes over time, and consequently we can draw conclusions different to those based on the data from the past. This is a highly dynamic situation, even for intensively studied organisms. For example, the human proteome in 2016 constituted 21 006 proteins with 71 173 splicing isoforms (92 179 in total). Now, we have 20 600 protein annotations with 79 500 splicing isoforms (100 100 in total), and this does not take into account the recent T2T-CHM13 reference genome update (43). The situation may be even more dramatic for proteomes that may have been only recently studied intensively in terms of proteomics. For example, X*enopus tropicalis* in 2016 had 18,252 annotated proteins, with an average isoelectric point of 6.70 and an average molecular mass of 60.1 kDa, accompanied by 5346 splicing isoforms (23 598 in total). Now, it has 22 514 proteins (average isoelectric point of 6.64 and average mass of 71.9 kDa), and 23 799 splicing isoforms have been identified. Accordingly, we decided to maintain the previous version of Proteome-*pI* (http://isoelectricpointdb.org) and present the new release as a completely new resource (http://isoelectricpointdb2.org).

### Future prospects

The number of reference proteomes has increased 4-fold during the last five years (5029 in Proteome-*pI* 1.0 versus 20 115 in the current release); therefore, constant addition of new proteomes is of great interest. Furthermore, users frequently request respective data for proteomes of interest to them, such as a particular strain of bacteria or virus not included in the official release but relevant to their ongoing studies (44). In parallel, the addition of new algorithms for isoelectric point and p*K*_a_ prediction is foreseen. The latter is especially worth consideration, as the database currently includes the prediction of p*K*_a_ values by only one method. This limitation will not be easy to overcome, as most of the p*K*_a_ predictors [e.g. Rosetta pKa (45), H++ (35), MCCE (36)] rely on protein structure information. However, the advance of the SWISS-MODEL Repository (46) and recently the AlphaFold Protein Structure Database (47) gives hope that Proteome-*pI* could be also extended by 3D-based protein predictions. It is worth mentioning here that there are already some efforts for making predictions of isoelectric points and p*K*_a_ values based on available protein structures [pKPDB database (48)]. Finally, one of the most important additions to the Proteome-*pI* database was introducing *in silico* proteome digests derived from the five most commonly used proteases. Furthermore, the resulting datasets were categorized by molecular mass to facilitate analysis with specific mass spectrometry techniques. Such an approach could be seen as highly simplistic, and further grinding of *in silico* digests is possible. Future plans in this respect include adding the prediction of peptides’ hydrophobicity, retention time (49), electrophoretic mobility (50), and the use of more sophisticated methods than can be utilized for the prediction of *in silico* digests [e.g. DeepDigest (51)]. Finally, adding information about the uniqueness of peptides versus coverage after digestion would be also valuable. We would be grateful for any contribution or ideas from the community with respect to future improvements to the database.

## DATA AVAILABILITY

All data in the Proteome-*pI* 2.0 database are available for download free of charge. For more information see Supplementary Data. The database will be maintained for at least 10 years and can be accessed at http://isoelectricpointdb2.org or http://isoelectricpointdb2.mimuw.edu.pl (mirror).

## Supplementary Material

gkab944_Supplemental_File
