# Supplementary material for: Proteome-pI 2.0: proteome isoelectric point database update
Source: Nucleic Acids Res. 2021 Oct 28;50(D1):D1535–40. doi: 10.1093/nar/gkab944 (PMC8728302; doi:10.1093/nar/gkab944)
Supplement: gkab944_Supplemental_File [file gkab944_supplemental_file.pdf]

Supplementary data

Proteome-*pI 2.0*: Proteome Isoelectric Point Database Update

Lukasz P. Kozlowski[1,*]

[1] Institute of Informatics, Faculty of Mathematics, Informatics, and Mechanics, University of Warsaw, Warsaw, Mazovian Voivodeship 02-097, Poland

* Corresponding author: lukasz.kozlowski.lpk@gmail.com

https://orcid.org/0000-0001-8187-1980

Database web address:

http://isoelectricpointdb2.org

http://isoelectricpointdb2.mimuw.edu.pl

Supplementary Figure 1. Isoelectric point predictions according to different methods. *Natronolimnobius baerhuensis:* archeaon living in soda lakes – top panel, *Danio rerio* – middle panel, *Methanothermus fervidus* thermophilic methanogen – bottom panel).

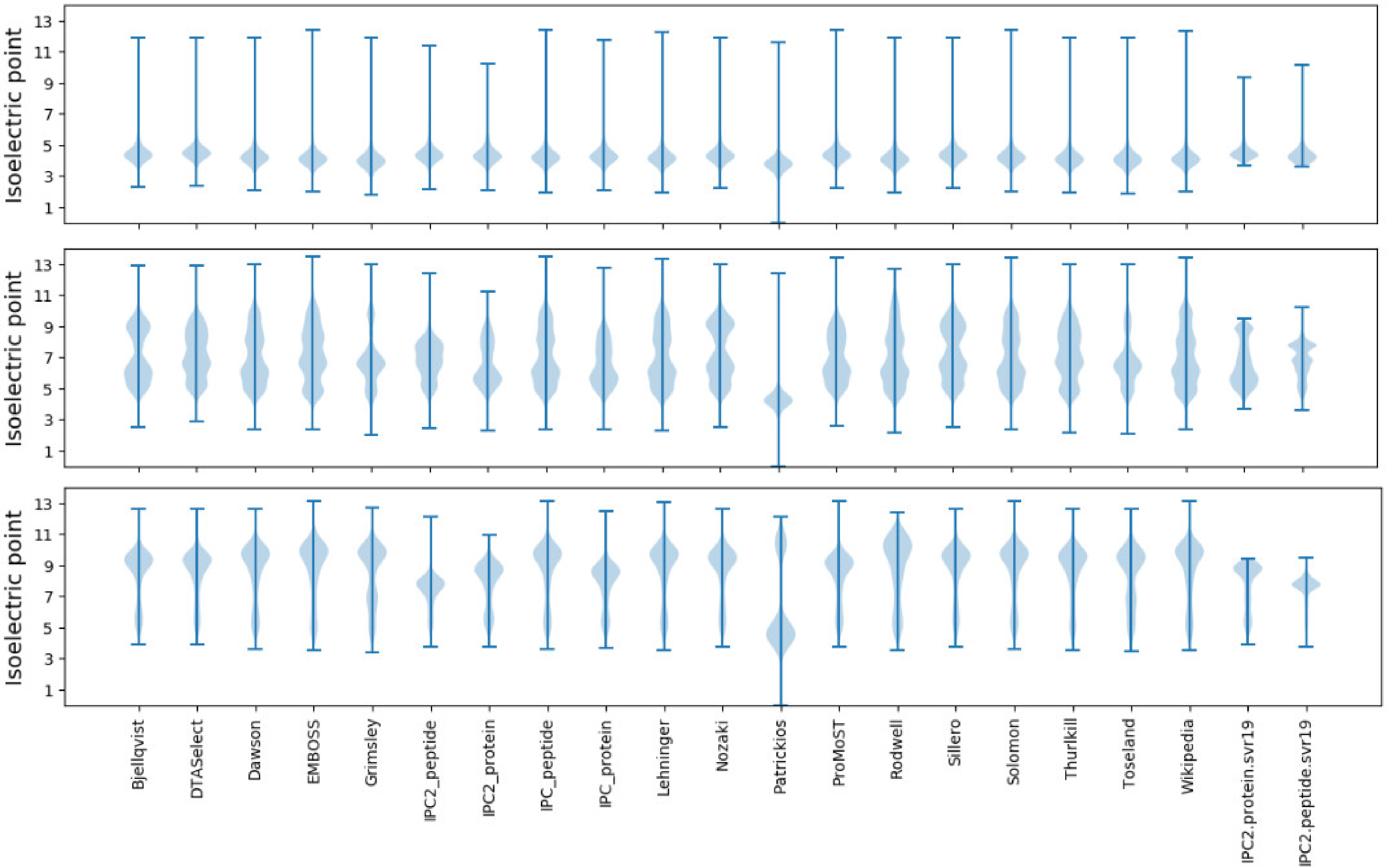

Supplementary Figure 2. Isoelectric points and molecular weights across the kingdoms of life: data for the proteomes of 331 Archaea, 4,046 viruses, 8,105 bacteria, and 1,612 eukaryota proteomes with at least 50 proteins.

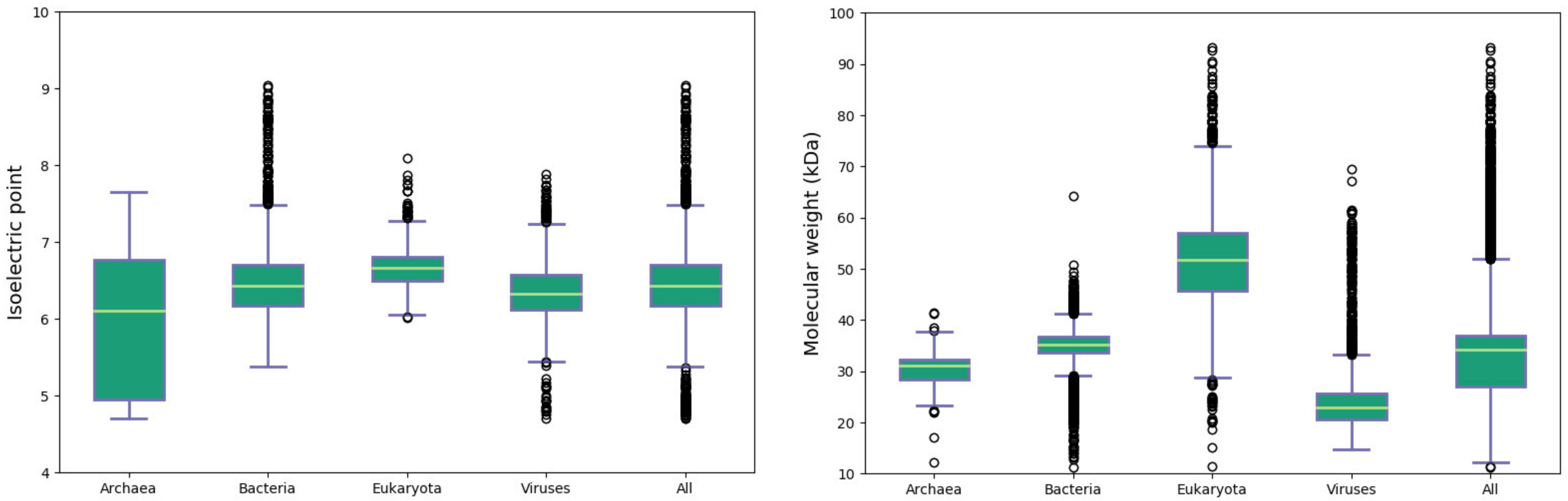

Supplementary Table S1. Selected statistics of the Proteome-*pI* 2.0 database

	IDM number of proteins	Median number of protein	IDM size of proteins	Median size of protein	IDM mw of proteins	Median mw of protein	Number of proteomes	Number of proteins
Viruses	35	17	182	145	20.4	16.4	10,064	518,140
Archaea	2,265	2,164	249	234	27.4	25.8	331	767,951
Bacteria	3,583	3,503	287	274	31.4	30.0	8,108	30,290,647
Eukaryote (all isoforms)	15,677	13,001	391	353	43.6	39.4	1,612	29,752,296
Eukaryote (main isoform)	14,121	12,791	370	336	41.2	37.5	1,612	25,437,198
Eukaryote (minor isoforms)	3,916	1,000	534	476	59.7	53.2	637	4,315,098

mw, molecular weight in kDa; IDM, interdecile mean.

Supplementary Table S2. *In silico* digestion of the human proteome (20,600 proteins and 79,500 splicing isoforms) with five proteases tailored for different mass spectrometry experiments (number of peptides).

	LTQ Orbitrap (600~4000 Da)	MALDI TOF/TOF (750~5500 Da)	ESI Ion Trap (600~3500 Da)	MS low (800~3500 Da)	MS high (600~5500 Da)
Trypsin	2,299,934	1,996,558	2,245,331	1,766,064	2,370,698
Chymotrypsin	1,747,732	1,651,584	1,680,404	1,410,810	1,858,042
Trypsin+LysC	2,363,493	2,021,295	2,312,300	1,794,859	2,427,011
LysN	1,385,317	1,357,297	1,314,283	1,109,938	1,516,346
ArgC	1,388,196	1,379,645	1,309,623	1,113,975	1,530,254

Supplementary Table S3. Di-amino acid frequency for Proteome-*pI* 2.0 (average from 20,115 proteomes)

	Ala	Cys	Asp	Glu	Phe	Gly	His	Ile	Lys	Leu	Met	Asn	Pro	Gln	Arg	Ser	Thr	Val	Trp	Tyr
Ala	10.31	1.14	4.59	5.49	3.10	6.60	1.77	4.25	3.78	8.79	1.87	2.68	4.22	3.23	5.12	5.96	4.74	6.33	1.05	2.11
Cys	1.03	0.35	0.77	0.79	0.61	1.17	0.40	0.75	0.70	1.41	0.27	0.56	0.80	0.56	0.87	1.20	0.77	0.95	0.20	0.42
Asp	4.67	0.75	3.52	3.98	2.24	4.21	1.20	3.07	2.52	5.29	1.12	1.94	2.96	1.82	3.06	3.59	2.72	3.81	0.78	1.69
Glu	5.53	0.79	3.69	5.51	2.09	3.89	1.41	3.51	4.04	6.07	1.43	2.66	2.60	2.70	4.08	3.79	3.32	4.12	0.75	1.66
Phe	2.98	0.65	2.29	2.17	1.67	2.86	0.90	2.04	1.72	3.70	0.73	1.53	1.75	1.37	1.94	3.01	2.17	2.56	0.52	1.22
Gly	5.89	1.02	3.74	4.12	2.85	5.94	1.66	3.75	3.62	6.52	1.55	2.58	3.05	2.54	4.30	5.20	4.02	4.85	1.04	2.18
His	1.76	0.43	1.16	1.25	0.96	1.68	0.82	1.19	0.97	2.38	0.45	0.82	1.46	0.98	1.48	1.66	1.22	1.48	0.32	0.73
Ile	4.46	0.84	3.09	3.28	2.04	3.56	1.21	2.90	2.69	4.79	0.98	2.20	2.70	1.94	2.78	3.89	2.99	3.50	0.59	1.54
Lys	3.93	0.66	2.80	3.80	1.61	3.01	1.13	2.83	3.77	4.63	1.07	2.27	2.44	2.08	3.01	3.36	2.82	3.19	0.58	1.51
Leu	8.84	1.45	5.36	5.96	3.60	6.42	2.32	4.64	4.96	9.89	1.87	3.57	5.34	4.06	5.93	7.28	5.32	6.34	1.12	2.51
Met	2.16	0.29	1.27	1.50	0.78	1.49	0.48	1.15	1.33	2.15	0.58	0.96	1.17	0.90	1.30	1.70	1.37	1.50	0.24	0.55
Asn	2.82	0.58	1.96	2.22	1.50	2.81	0.88	2.34	2.04	3.62	0.80	1.95	2.14	1.50	1.96	2.78	2.09	2.44	0.49	1.23
Pro	4.81	0.63	2.98	3.58	1.87	3.87	1.20	2.24	2.25	4.60	0.92	1.81	3.84	2.05	2.77	4.31	2.99	3.71	0.63	1.38
Gln	3.36	0.53	1.86	2.52	1.32	2.39	1.02	2.02	2.09	3.91	0.86	1.56	2.08	2.57	2.53	2.61	2.08	2.50	0.51	1.07
Arg	4.90	0.83	3.14	3.80	2.22	3.74	1.50	3.05	3.07	5.91	1.21	2.12	2.98	2.40	4.66	4.00	3.06	3.75	0.84	1.68
Ser	5.78	1.12	3.79	4.04	2.91	5.43	1.67	3.66	3.45	7.00	1.42	2.81	4.22	2.77	4.06	7.21	4.30	4.67	0.93	2.00
Thr	4.98	0.82	2.89	3.19	2.13	4.24	1.21	2.96	2.45	5.43	1.08	2.00	3.42	1.92	2.84	4.24	3.59	4.13	0.70	1.53
Val	6.12	1.04	3.85	4.24	2.62	4.41	1.50	3.54	3.20	6.71	1.38	2.46	3.51	2.40	3.82	4.78	4.00	5.17	0.82	1.83
Trp	0.95	0.20	0.69	0.71	0.51	0.79	0.31	0.67	0.68	1.36	0.31	0.55	0.53	0.55	0.88	0.88	0.73	0.79	0.23	0.36
Tyr	2.09	0.49	1.63	1.65	1.27	2.05	0.71	1.48	1.36	2.79	0.55	1.21	1.32	1.14	1.71	1.98	1.54	1.77	0.39	1.01

Similar statistics for 20,115 individual proteomes and for grouped proteomes from different kingdoms of life are available online on individual subpages of the database and in Statistics section, respectively.

All values are presented as per mille (‰) and therefore need to be multiplied by 10^{-3}. For better visibility, di-amino acids that are more frequent than expected are marked in red, and those that are underrepresented are marked in blue. Order in the table: RowColumn (e.g., AlaCys 1.14, CysAla 1.03).

Availability of the raw data from Proteome-*pI* 2.0

Most of the data available in Proteome-*pI* 2.0 are available to download in either CSV or fasta format. The header line for fasta files has been enriched with information about isoelectric point (proteins and peptides) and dissociation constant (pK_a) predictions (proteins):

- to download isoelectric points, pK_a values and molecular weights for **proteins** (all 21 *pI* methods) of individual proteomes, see: http://isoelectricpointdb2.mimuw.edu.pl/protein-download-instruction.txt
- to download isoelectric points and molecular weights for **peptides** (all five digests) of individual proteomes, see: http://isoelectricpointdb2.mimuw.edu.pl/peptide-download-instruction.txt

For high-throughput analysis, isoelectric points predicted for big protein databases are also available:

- non-redundant (nr) NCBI proteins (409 million proteins) June 2021. RepOD. http://dx.doi.org/10.18150/CPEQGE
- UniProtKB/TrEMBL proteins (219 million proteins) June 2021. RepOD. http://dx.doi.org/10.18150/EARKJV
- SwissProt proteins (~561,000 proteins) June 2021. RepOD. http://dx.doi.org/10.18150/GVTHDK
- Protein Data Bank proteins (~601,000 protein chains) June 2021. RepOD. http://dx.doi.org/10.18150/SGEKFJ
- 250,000 of proteins with the lowest isoelectric points http://isoelectricpointdb2.mimuw.edu.pl/extra_large_db/proteins_with_lowest_isoelectric_point_250k.fasta
- 250,000 of proteins with the highest isoelectric points http://isoelectricpointdb2.mimuw.edu.pl/extra_large_db/proteins_with_highest_isoelectric_point_250k.fasta

For cumulative statistics (e.g., average isoelectric points), see: http://isoelectricpointdb2.mimuw.edu.pl/general-statistics-proteome-pi.csv
